# Enhanced rice salinity tolerance via CRISPR/Cas9-targeted mutagenesis of the *OsRR22* gene

**DOI:** 10.1007/s11032-019-0954-y

**Published:** 2019-03-09

**Authors:** Anning Zhang, Yi Liu, Feiming Wang, Tianfei Li, Zhihao Chen, Deyan Kong, Junguo Bi, Fenyun Zhang, Xingxing Luo, Jiahong Wang, Jinjuan Tang, Xinqiao Yu, Guolan Liu, Lijun Luo

**Affiliations:** 1 Huazhong Agricultural University, Wuhan 430070, People’s Republic of China; 2 Shanghai Agrobiological Gene Center, Shanghai 201106, People’s Republic of China

**Keywords:** CRISPR/Cas9, *OsRR22*, Salinity tolerance, Genetic engineering, Abiotic stress

## Abstract

Salinity is one of the most important abiotic stress affecting the world rice production. The cultivation of salinity-tolerant cultivars is the most cost-effective and environmentally friendly approach for salinity control. In recent years, CRISPR/Cas9 systems have been widely used for target-site genome editing; however, their application for the improvement of elite rice cultivars has rarely been reported. Here, we report the improvement of the rice salinity tolerance by engineering a Cas9-OsRR22-gRNA expressing vector, targeting the *OsRR22* gene in rice. Nine mutant plants were identified from 14 T_0_ transgenic plants. Sequencing showed that these plants had six mutation types at the target site, all of which were successfully transmitted to the next generations. Mutant plants without transferred DNA (T-DNA) were obtained via segregation in the T1 generations. Two T2 homozygous mutant lines were further examined for their salinity tolerance and agronomic traits. The results showed that, at the seedling stage, the salinity tolerance of T2 homozygous mutant lines was significantly enhanced compared to wild-type plants. Furthermore, no significantly different agronomic traits were found between T2 homozygous mutant lines and wild-type plants. Our results indicate CRISPR/ Cas9 as a useful approach to enhance the salinity tolerance of rice.

## Introduction

The global crop production needs to double by 2050 to match the demands of the rapidly increasing population, changing diet, and increasing biofuel consumption (Ray et al. 2013). However, abiotic stress, which includes drought, salinity, low temperature, heat, flooding, and oxidative stress, severely limits the feasible yield increase, or even reduces crop production in large areas (Mahajan and Tuteja 2005). Among these abiotic stress, salinity poses one of the major threats to crop production since most crop plants cannot grow under a high concentration of salt (Munns and Tester 2008). Furthermore, soil salinity is hard to remove, which will cause a continuous decrease in crop production for many years. Over 400 million hectares of land throughout the world have been affected by salinity (http://www.plantstress.com/Articles/index.asp). Moreover, land affected by salt stress is arising due to various factors such as climate change, sea-level increases, and tsunamis (Kumar et al. 2013). Consequently, salinity remains a severe threat to the food supply.

Rice (*Oryza saliva* L.) is one of the most important food crops and forms the main staple food for more than half of the world’s population. Since rice is a species originally grown in swamps and freshwater marshes, it is particularly sensitive to salt stress and rated as a particularly salt-sensitive crop (Dionisio-Sese and Tobita 1998; Kumar et al. 2013). Salinity is one of the major obstacles for rice production especially at the seedling stage (Lutts et al. 1995). Researching of rice salt tolerance is becoming increasingly urgent and improving the salt tolerance of rice has become an important breeding goal. Numerous salt tolerance quantitative trait loci were identified and few of them had been transferred into popular rice varieties via marker-assisted selection (MAS) (Lang et al. 2011; Bimpong et al. 2016; Jing and Zhang. 2017). During the past two decades, many salt-related genes *(SKC1, DST, OsRR22, OsHAL3, P5CS, SNAC2,* and *OsNAP*) have been successfully cloned (Ren et al. 2005; Hu et al. 2008; Huang et al. 2009; Sun et al. 2009; Karthikeyan et al. 2011; Chen et al. 2014; Takagi et al. 2015). Among them, the *OsRR22* gene encodes a 696-amino acid B-type response regulator transcription factor that is involved in both cytokinin signal transduction and metabolism; its loss of function has been reported to significantly increase salt tolerance (Takagi et al. 2015).

The CRISPR/Cas9 system is an accurate, convenient, and efficient genome-editing method developed during recent years (Shan et al. 2013). At present, the CRISPR/Cas9 system has been widely used for genome editing in major crops such as wheat (Wang et al. 2014; Liang et al. 2017), maize (Svitashev et al. 2016; Zhu et al. 2016), and sorghum (Li et al. 2015; Cai et al. 2015). In rice, using CRISPR/Cas9 technology, many genes (*OsPDS, OsERF922, OsHAK1, Badh2, and TMS5*) have been knocked out and the expected phenotype was obtained (Zhang et al. 2014; Wang et al. 2016; Zhou et al. 2016; Nieves-Cordones et al. 2017; Shao et al. 2017). This system provides a new method for rice breeding. This study first reports the improvement of salinity tolerance via CRISPR/Cas9-targeted mutagenesis of the transcription factor OsRR22.

## Materials and methods

### Plant growth conditions

The elite *japonica* rice cultivar WPB106 was bred from ‘Huhan9/Huxiangjing//Huhan3/Huhan11’ in our laboratory. All transgenic plants and WPB106 (wild type, WT) were grown in the greenhouse at 28–35 °C, in Shanghai, or in fields at the station of the Shanghai Academy of Agricultural Sciences under normal growth conditions. For salinity stress at the seedling stage, seedlings of rice were cultivated in normal nutrient solution for 5 days after germination on a 96-well plate (Xia et al. 2017). They were placed in a growth chamber (14 h of daytime at 30 °C and 10 hat night at 20 °C with 70% relative humidity).

### Vector construction

**Fig. 1:**
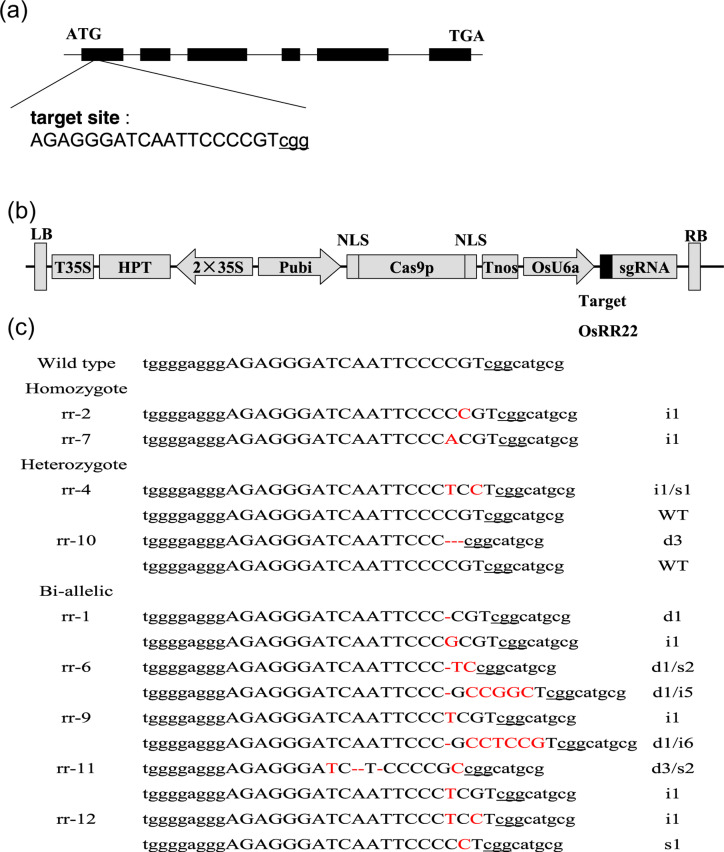
CRISPR/Cas9-induced *OsRR22* gene modification in rice. **a** Schematic of the *OsRR22* gene structure and target site. Exons and introns are indicated with black rectangles and black lines, respectively. Both the translation initiation codon (ATG) and the termination codon (TGA) are shown. The target site nucleotides are shown in capital letters and the protospacer adjacent motif (PAM) site is underlined. **b** Schematic presentation of the T-DNA structure in the CRISPR/Cas9-mediated genome editing construct. The expression of Cas9 is driven by the maize ubiquitin promoter (Pubi); the expression of the sgRNA scaffold is driven by the rice U6a small nuclear RNA promoter (OsU6a); the expression of hygromycin (HPT) is driven by two CaMV35S promoters (2 × 35S). Abbreviations: NLS, nuclear localization signal; Tnos, gene terminator; LB and RB, left border and right border, respectively. **c** Nucleotide sequences at the target site in the nine T_0_ mutant rice plants. The recovered mutated alleles are shown below the wild-type sequence. The target site nucleotides are indicated with black capital letters. The PAM site is underlined. The red dashes indicate deleted nucleotides. The red capital letters indicate inserted or substituted nucleotides. The numbers on the right indicate the type ofmutation and the number of nucleotides involved. “i,” “d,” and “s” indicate insertion, deletion, and substitution of the indicated number of nucleotides, respectively; “WT” indicates wild-type

The Cas9 plant expression vector (pYLCRISPR/ Cas9Pubi-H) and the sgRNA expression vector (pYLgRNA) were provided by Prof. Yao-Guang Liu of the South China Agricultural University. The Cas9-OsRR22-gRNA expressing vector was constructed following previously described protocol (Ma et al. 2015a). Briefly, according to the design principles of the target sequences of the CRISPR/ Cas9 system, 19 to 20 bases upstream of the protospacer adjacent motif (PAM) were selected as candidate target sequence (Fig. 1 a). A BLAST search (http://blast.ncbi.nlm.nih.gov/Blast.cgi) of the target sequences (including PAM) against the rice genome was conducted to confirm their targeting specificity in the genome. The target sequence has a difference of at least two bases compared with similar non-target sequences within the PAM or PAM-proximal region. The gRNA expression cassette was synthesized via overlapping PCR. The target-specific sequence of gRNA (target *OsRR22*) was put at the 5’-end of the primers RR22-gRT+/RR22-OsU6aT-. Two PCR reactions were performed, using the plasmid pYLgRNA-OsU6a/LacZ as template. The first PCR was performed using the primer set U-F/RR22-OsU6aT-, and the second one used the primer set RR22-gRT+/gR-R (Table 1). The products of PCR 1 and 2 were used as templates for the third PCR reaction with the primer set U-GAL/Pgs-GAR to generate the full-length gRNA fragment (Table 1). Subsequently, amplicons containing OsRR22-gRNA with different *Bsal-cutting* sites were cloned into the Cas9 plant expression vector pYLCRISPR/Cas9Pubi-H at the *Bsal* site, using the pEASY-Uni Seamless Cloning and Assembly Kit (TransGen Biotech, Beijing, China). The resultant construct Cas9-OsRR22-gRNA contained a Cas9p expression cassette (Pubi::NLS::Cas9 p::NLS::Tnos) and a hygromycin resistance cassette (2xP35S::HPT::T35STnos) (Fig. 1 b).

**Table 1:** Primers used in this study

Primer name Primer	sequence (5′-3′)	Purpose
RR22-gRT+	AGAGGGATCAATTCCCCGTgttttagagctagaaat	Vector construct
RR22-OsU6aT-	ACGGGGAATTGATCCCTCTCggcagccaagccagca	Vector construct
U-F	CTCCGTTTTACCTGTGGAATCG	Vector construct
gR-R	CGGAGGAAAATTCCATCCAC	Vector construct
U-GAL	ACCGGTAAGGCGCGCCGTAGTGCTCGACTAGTATGGAATCGGCAGCAAAGG	Vector construct
Pgs-GAR	TAGCTCGAGAGGCGCGCCAATGATACCGACGCGTATCCATCCACTCCAAGCTCTTG	Vector construct
RR22-S-F	CTTGGGATTGCTCTGTTTCT	Target site sequencing
RR22-S-R	GTAATAGCCTGGTTGGTTGAT	Target site sequencing
HPT-F	GCTCCATACAAGCCAACCACG	Transgenic analysis
HPT-R	CCTGCCTGAAACCGAACTGC	Transgenic analysis
CAS9-F	CGAGACGAACGGTGAGACTGGTG	Transgenic analysis
CAS9-R	GGTGCTTGTTGTAGGCGGAGAGG	Transgenic analysis

**Fig. 2:**
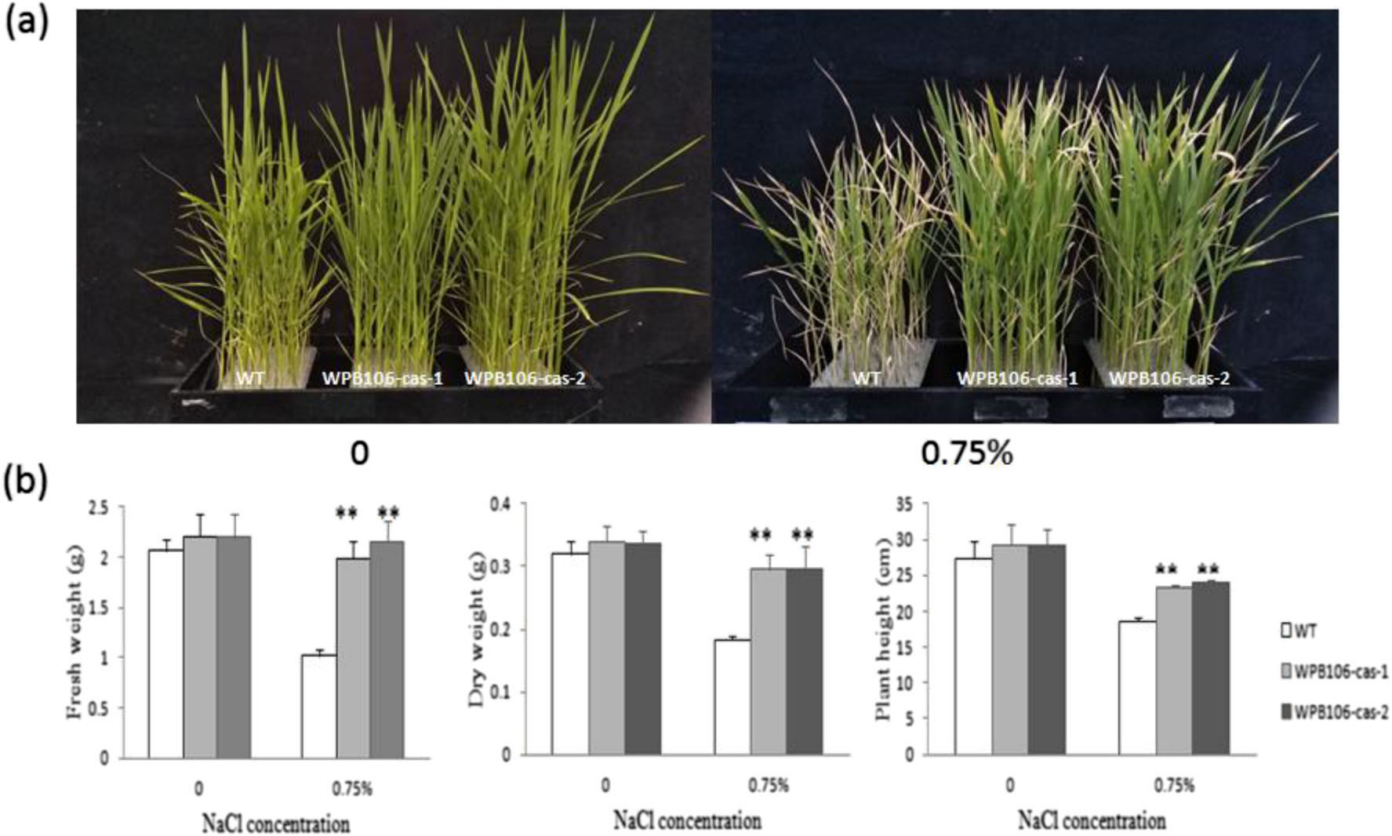
Identification of salinity tolerance in homozygous mutant rice lines. a Phenotypes of 4-week-old WT, WPB106-cas-1, and WPB106-cas-2 plants grown with underground fresh water and subjected to a concentration of 0.75% NaCl. Two-week-old plants were treated with concentrations of 0.75% NaCl. Then, phenotypic evaluation was conducted 14 days after treatment. b A comparison of shoot fresh weight, shoot dry weight, and plant height between WT, WPB106-cas-1, and WPB106-cas-2 plants is shown in a. Values of shoot fresh weight and shoot dry weight represent weight of 10 plants per treatment. Mean values and standard deviations are shown. Asterisks indicate significant differences to WT (**P <0 0.01)

### *Agrobaclerium*-mediated rice transformation

The Cas9-OsRR22-gRNA expressing binary vector was introduced into the japonica rice WPB106 cultivar via the *Agrobaclerium*-mediated transformation method described by Nishimura et al. (2006). Hygromycin-containing medium was used to select hygromycin-resistant calli, and then, vigorously growing calli were transferred to regeneration media to generate green plants.

### Identification of mutant transgenic plants

To determine the mutation at the target site, genomic DNA from the leaves of transgenic plants was extracted using a DNA Quick Plant System (TransGen Biotech, Beijing, China). Genomic DNA (50 ng) was used as template to perform PCR amplification using PCR Mastermix (TIANGEN, Beijing, China). PCR was performed to amplify the genomic region containing the CRISPR/Cas9 target site, using specific primer pairs that surround the designed target site (Table 1). The PCR products were directly sequenced or cloned into the pEASY-Blunt vector (TransGen Biotech, Beijing, China) and sequenced using the Sanger method. Mutations were identified by comparing the sequences of transgenic plants with those of WT plants. Mutations containing normal sequencing chromatograms were considered as homozygote mutations. Mutations containing superimposed sequencing chromatograms were considered heterozygous or bi-allelicmutations, which were decoded via degenerate sequence decoding (Ma et al. 2015b).

To identify T-DNA-free plants from T_1_, the plants were analyzed via PCR using HPT-specific and *Cas9-* specific primers (Table 1) in combination with agarose gel electrophoresis. The pYLCRISPR/Cas9Pubi-H plasmids and the T_0_ transgenic plants were selected as positive controls and WPB106 DNA and H_2_O were used as negative controls. *HPT-* and *Cas9*-negative plants were considered as T-DNA-free plants.

### Greenhouse trials for salinity tolerance

To evaluate the salinity tolerance of plants at the seedling stage, a salt stress test was performed according to the method published by Takagi et al. (2015). We compared the salinity tolerance of 2-week-old WT and homozygous mutant plants in the greenhouse of the Shanghai Agrobiological Gene Center, Shanghai. Briefly, 2-week-old plants were treated with fresh groundwater and concentrations of 0.75% NaCl solution (pH = 7), respectively. After 2 weeks of treatment, the salinity tolerance was determined via plant height and shoot fresh and dry weights of 10 plants per line. Each line was replicated three times.

### Evaluation of major agronomic traits under field conditions

To evaluate the agronomic traits under normal field conditions, both WT and mutant plants were planted in a four-row plot with seven plants per row, applying 20 x 15 cm spacing in Shanghai, China, during the summer of2017. In these field trials, 4-week-old plants (or older) were transplanted and field management was conducted according to local conventional methods. The agronomic traits were measured according to the SES (IRRI 2002). Five plants in the middle row of each line were sampled for the following agronomic traits: days to 50% flowering, plant height, no. of tillers, no. of grains per panicle, spikelet fertility, 1000-seed weight, and yield per plant.

## Results

### CRISPR/Cas9 design

To design a mutation specifically targeting the *OsRR22* gene in rice, a 20-bp nucleotide sequence in the first exon of *OsRR22* (GenBank Accession No BR000251.1) was chosen as the target site (Fig. 1 a). The binary plasmid Cas9-OsRR22-gRNA (Fig. 1 b) was constructed based on the CRISPR/Cas9 vector previously described by Ma et al. (2015a). The vector was used to transform the rice variety WPB106 via *Agrobacterium-medi*ated transformation. Using site-specific PCR and Sanger sequencing, a total of nine WPB106 mutants were recovered from 14 T_0_ hygromycin-resistant transgenic WPB106 plants (64.3%). These nine mutants were detected and then subjected to zygosity analysis by cloning PCR products into the T vector for DNA sequencing. Sequence analyses detected two homozygous mutations, two heterozygous mutations, and five bi-allelic mutations. Based on allele mutation types, 44.4% of the mutations were nucleotide insertions, 11.1% of the mutations were nucleotide deletions, and 5.6% of the mutations were nucleotide substitutions; 11.1%, 5.6%, and 11.1% of the mutations were simultaneous nucleotide insertions and deletions, insertions and substitutions, and deletions and substitutions, respectively (Table 2).

**Table 2:** Ratios of mutant genotype and mutation type at the target site in T0 mutant plants

Mutant genotype ratios (%) ^a^			Mutation type ratios (%) ^b^					
Bi-allele	Homozygote	Heterozygote	Deletion	Insertion	Substitution	Insertion and deletion	Insertion and substitution	Deletion and substitution
55.6 (5/9)	22.2 (2/9)	22.2 (2/9)	11.1 (2/18)	44.4 (8/18)	5.6 (1/18)	11.1 (2/18)	5.6 (1/18)	11.1 (2/18)

a Based on the number of each mutant genotype out of the total number of all mutant genotypes at the target site

b Based on the number of each allele mutation type out of the total number of all allele mutation types at the target site

### Transmission of CRISPR/Cas9-induced mutations to the T_1_ generation

To further understand the inheritance of the mutation, two homozygous (rr-2, rr-7), one heterozygous (rr-10), and one bi-allelic (rr-1) T_0_ mutant plants (Fig. 1 c) were self-pollinated, and their progenies were genotyped at the target site. We randomly selected nine to 23 T_1_ progenies derived from each T_0_ plant for genotyping analysis (Table 3). As expected, all of these T_0_ putative homozygotes and their offspring had identical genotypes (rr-2 and rr-7), suggesting stable transmittance of the mutations in these homozygous mutant lines to the next generation. Bi-allelic mutations in T_0_ plants were apparently transmitted to the T_1_ generation following the Mendelian genetic law, indicating that the targeted mutations in T_0_ plants were inherited normally. For example, the bi-allelic T_0_ mutant plant rr-1 harbors two mutations (a 1-bp deletion (d1) and a 1-bp insertion (i1)); its T_1_ progenies segregated in a ratio of 5 (d1):8 (d1/i1):4 (i1), which is consistent with the predicted Mendelian segregation (*χ*^2^ = 0.176 < *χ*^2^_0.05,2_ = 5.99) (Table 3). For the T_1_ generation of heterozygous (rr-10), several new mutations (6 (d1/i1)) were generated. In combination, these results clearly demonstrated that CRISPR/Cas9-induced gene mutations could be stably transmitted to subsequent generations.

**Table 3:** CRISPR/Cas9-induced mutations in *OsRR22* and their transmission to the T_1_ generation

T_0_ plant	Genotype	Mutation type	Number of T_1_ plants tested	Mutation transmission in the T_1_ generation		No. of T-DNA-free plants
				Targeted mutations	*χ*^2^ (1:2:1)	
rr-2	Homozygote	i1	10	10 (i1)	ND	2
rr-7 Homozygote	i1	9	9 (i1)	ND	3
rr-10	Heterozygote	d3	23	5 (d3), 8 (d3/wt), 4 (wt), 6 (d1/i1)	ND	0
rr-1	Bi-allelic	d1/i1	17	5 (d1), 8 (d1/i1), 4 (i1)	0.176 (*P* > 0.05)	4

“i” and “Bd” indicate insertion and deletion of the indicated number of nucleotides, respectively; “d/i” indicates the simultaneous deletion and insertion of the indicated number of nucleotides. The numbers on the right indicate the type of mutation and the number of nucleotides involved *WT*, wild type; *ND*, not detected

### Selection of T-DNA-free mutant rice lines

To obtain rice lines harboring the desired *OsRR22* mutations without T-DNA of the construct Cas9-OsRR22, we conducted PCR amplification using the primer sets designed to amplify Cas9 and HPT sequences (Table 1). The absence of transgenes was determined via negative PCR results of both Cas9 and HPT. T-DNA-free plants were found among most T_1_ plants, with the proportion ranging from 20.0 to 33.3% (Table 3). These results indicated that T-DNA-free homozygous mutants could be acquired via segregation populations. We isolated two T-DNA-free homozygous mutant lines (rr-2-1 and rr7-4) in the T1 generation to produce the T2 population to identify the salinity-tolerant phenotypes, designated as WPB106-cas-1 and WPB106-cas-2.

### Salinity tolerance was enhanced in OsRR22-induced mutations

To evaluate the salinity-tolerant phenotype of the obtained rice mutants, two homozygous mutant T2 lines (WPB106-cas-1 and WPB106-cas-2) with different allelic mutations and WT plants were treated with fresh groundwater and a concentration of 0.75% NaCl nutrition solution at the 2-week-old stage. Compared to WT, two mutant lines grew better than WT under this condition (Fig. 2 a). As measured after 2 weeks of treatment, the shoot fresh weight of WT was reduced by 50.3%, while WPB106-cas-1 and WPB106-cas-2 showed only 10.1% and 2.1% reduction in shoot fresh weight, compared to plants that were grown with fresh groundwater. Similarly, the shoot dry weight of WT had been reduced by 42.6%, whereas WPB106-cas-1 and WPB106-cas-2 showed only decreases of 12.9% and 12.3%. The 0.75% NaCl treatment also caused decreases of 31.8%, 20.3%, and 17.8% in plant height of WT, WPB106-cas-1, and WPB106-cas-2, respectively (Fig. 2 b and Table S1). The significant difference analysis of the shoot fresh weight, shoot dry weight, and plant height indicated that two mutant lines were significantly different from WT plants. These results implicitly indicate that CRISPR/ Cas9-induced mutations in the *OsRR22* gene enhanced the tolerance to salinity.

### The main agronomic traits were not altered in rice mutants

To survey whether mutations in the *OsRR22* gene affect other agronomic traits, we characterized two homozygous T_2_ mutant lines by measuring their plant height, days to 50% flowering, no. of tillers per plant, no. of grains per panicle, spikelet fertility, 1000-seed weight, and yield per plant under normal field conditions. Student’s *t* test showed that none of the T_2_ mutant lines was significantly different from WT plants under normal growth conditions (Table 4). These results showed that CRISPR/Cas9-induced mutations in the *OsRR22* gene did not significantly influence agronomic traits under normal field conditions.

**Table 4:** Agronomic traits of homozygous T2 mutant lines

Lines	Days to 50% flowering	Plant height (cm)	No. of tillers per plant	No. of grains per panicle	Spikelet fertility (%)	1000-seed weight (g)	Yield per plant (g)
WT	87.81 ± 0.7a	92.82 ± 3.91a	8.49 ± 0.25a	123.51 ± 3.23a	93.11 ± 3.09a	25.15 ± 0.79a	21.19 ± 0.57a
WPB106-cas-1	87.01 ± 1.19a	95.01 ± 2.23a	8.43 ± 0.21a	124.01 ± 4.46a	96.45 ± 1.98a	25.05 ± 0.94a	20.72 ± 0.77a
WPB106-cas-2	86.51 ± 0.71a	93.03 ± 2.86a	8.38 ± 0.23a	123.52 ± 3.05a	94.29 ± 2.58a	25.29 ± 0.61a	20.82 ± 0.64a

The data are measured for five plants per line. Values followed by the same letter (a) are not significantly different (P < 0.05)

## Discussion

CRISPR/Cas9 is a new genome-editing technique, which is highly specific and efficient. So far, the CRISPR/Cas9 technology has been widely used to improve major crops, such as rape, corn, rice, and soybean (Bortesi and Fischer 2015). However, few studies reported the direct genome editing of elite rice cultivars with the CRISPR/Cas9 technology. The ERF transcription factor gene *OsERF922* was mutated by CRISPR/ Cas9 to enhance the blast resistance of the rice variety Kuiku131 with normal phenotypes (Wang et al. 2016). Knockout of the thermo-sensitive genic male-sterile (TGMS) gene *tms5* of 11 fertile elite cultivars produced TGMS lines with good agronomic characteristics (Zhou et al. 2016). WPB106, a water-saving and drought-resistant elite japonica cultivar (Luo 2010), has the advantages of drought resistance, early maturity, and cooking quality; however, it is very sensitive to salinity. To quickly improve its salt tolerance, we applied the CRISPR/Cas9 technology. In this study, we used the Cas9-OsRR22-gRNA expressing vector to knockout *OsRR22* and achieved 64.3% mutant plants in T_0_ transgenic plants. We obtained two homozygous mutant lines that harbor mutagenesis in *OsRR22* without exogenous T-DNA. The evaluation of the salinity tolerance at the seedling stage showed that the salinity tolerance of T_2_ homozygous mutant lines was significantly enhanced compared to that of WT plants. Furthermore, the result of field trials showed no significant difference between T_2_ homozygous mutant lines and WT plants in the main agronomic traits under normal field conditions. Our study provides a successful case for improving rice salinity tolerance via the CRISPR/Cas9 technology and thus demonstrated that *OsRR22* has promising potential to accelerate the improvement of the salinity tolerance in rice breeding.

Many genes involved with salinity tolerance have been identified in rice, such as *OsNAC6, OsPPla, OsTPSl,* and *OsNA’P.* Transgenic rice plants overexpressing these genes showed an improved tolerance to high salt stresses. However, transgenic plants generated by gene addition are subjected to rigorous genetically modified management. Breeding strategy using CRISPR/Cas9 technology knockdown of rice transcription factor has been demonstrated to be an alternative approach for genetic improvement of rice and avoiding transgenic issue. According to the reports, there are only a few genes acting as a negative regulator of salt tolerance. Although the DST knockdown mutant could effectively improve salt tolerance, it has a large change in agronomic traits, such as leaf width, the panicle number per plant, and the main panicle length (Huang et al. 2009). OsRR22 could significantly improve salt tolerance but no changes were found in other agronomic traits (Takagi et al. 2015). Through our experiments, improved salt tolerance in the OsRR22 knockout lines free of transgene has been verified. Similarly, there was no alteration in the agronomic traits under normal conditions, which has achieved our breeding goals. Salinity tolerance is usually related to drought tolerance. Interestingly, we also carried out the drought-tolerant identification of WT and two homozygous T_2_ mutant lines at the seedling stage, and the results showed that there was no difference between WT and mutant lines under drought stress (Fig. S1).

In the present study, the Cas9-OsRR22-gRNA-induced mutagenic frequency of T_0_ plants was 64.3% and the homozygous rate of T0 mutant plants was 22.2%, which was similar to previous reported values in rice (Zhang et al. 2014; Wang et al. 2016; Zhou et al. 2016). Among six types of induced mutations in T_0_ plants, single-nucleotide insertions were most frequently detected (up to 44.4%), which is consistent with a previous report (Zhang et al. 2014). In addition, allele mutations could be successfully transmitted to the next generations. Moreover, we observed new mutations within the T_1_ offspring of rr-10, which are probably due to the continuous modification of WT alleles in Cas9-positive T_1_ lines. T-DNA-free plants could be found in almost all T_1_ segregation population. These results indicate a very convenient production of T-DNA-free homozygous mutation lines in the T_1_ generation.

In conventional rice breeding, efforts to breed for salinity tolerance have been attempted. However, these usually required approximately one decade due to the lack of accurate screening techniques, lack of adequate resistance resources, and timeconsuming backcrossing procedure (Hoang et al. 2016). Compared to conventional breeding, the CRISPR/Cas9 technology offers the ability to shorten the breeding period and thus significantly reducing cost (Schaart et al. 2016). For example, this experiment showed that we only required 1 year to improve the salt tolerance of WPB106 via CRISPR/ Cas9 technology. Furthermore, the CRISPR/Cas9 technology is more accurate than conventional breeding, since it only creates mutations in the target gene without changing other genes. However, rarely, negative regulatory genes with the desired function and the requirement for a PAM (-NGG) sequence form limitations of the CRISPR/Cas9 system. Conventional breeding has the advantage to improve the complex trait, while the CRISPR/Cas9 technique has the advantage in the mutagenesis of key genes. Therefore, the present study indicates that combining the CRISPR/Cas9 technique with conventional rice breeding could become a very powerful new tool for crop improvement.
