# Supplementary material for: Enhanced rice salinity tolerance via CRISPR/Cas9-targeted mutagenesis of the OsRR22 gene
Source: Mol Breed. 2019 Mar 9;39:47. doi: 10.1007/s11032-019-0954-y (PMC7413041; doi:10.1007/s11032-019-0954-y)
Supplement: Supplementary file 1 [file MB-2019-s11032-019-0954-y-S1.docx]

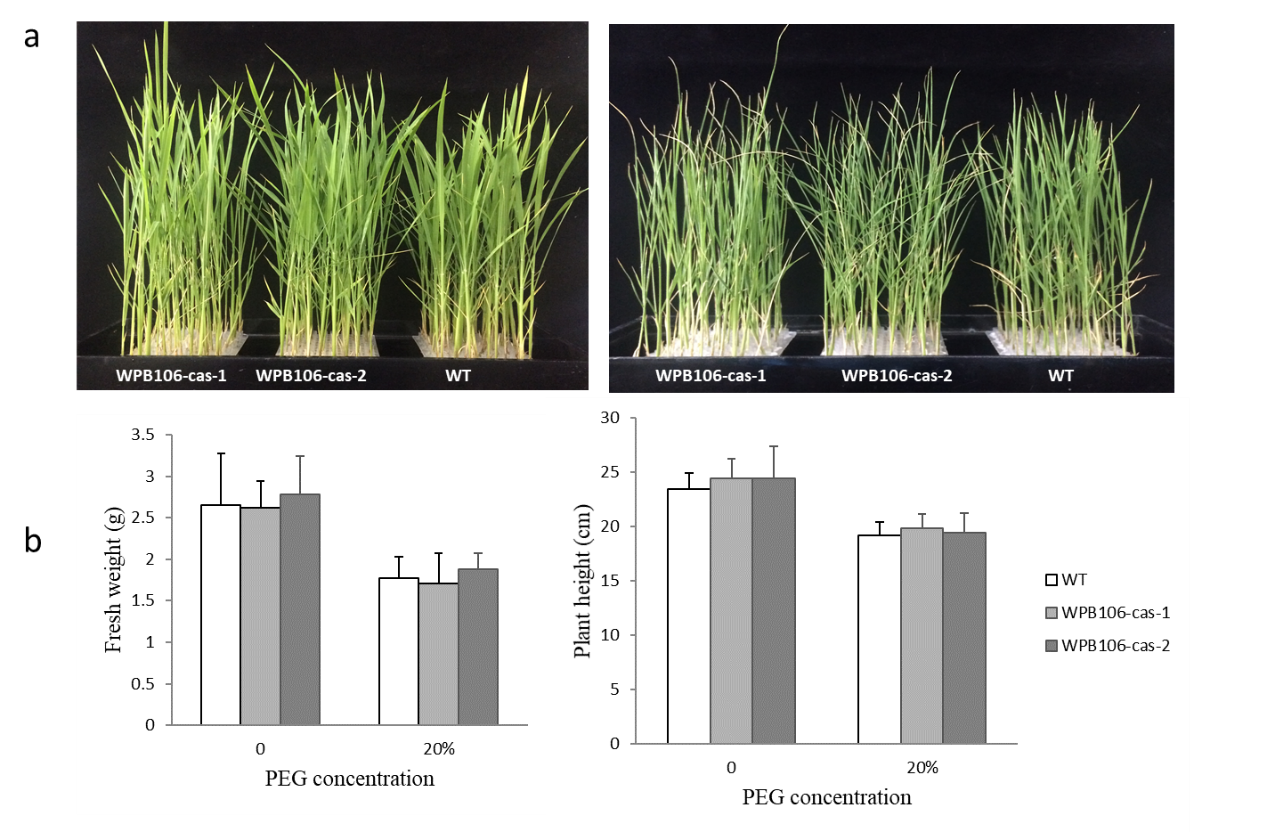


Fig. S1 Identification of drought tolerance of homozygous T2 mutant lines. (a) Phenotypes of four-week-old WT, WPB106-cas-1 and WPB106-cas-2 plants grown at underground fresh water and concentration of 20% PEG. Three-week-old plants were treated with concentrations of 20% PEG. Then, phenotypic evaluation was done 7 days after treatment. (b) Comparison of shoot fresh weight and plant height of WT, WPB106-cas-1 and WPB106-cas-2 plants shown in a. Values of shoot fresh weight represent weight of ten plants in each treatment. Mean values and standard deviations are shown. Asterisks indicate significant difference with WT (***P* < 0.01).
