# Supplementary material for: Enhanced rice salinity tolerance via CRISPR/Cas9-targeted mutagenesis of the OsRR22 gene
Source: Mol Breed. 2019 Mar 9;39:47. doi: 10.1007/s11032-019-0954-y (PMC7413041; doi:10.1007/s11032-019-0954-y)
Supplement: Supplementary file 2 [file MB-2019-s11032-019-0954-y-S2.docx]

| Table S1 Performance of salinity tolerance of homozygous T2 mutant lines | | | | | | | | |
| --- | --- | --- | --- | --- | --- | --- | --- | --- |
| Rice lines | Shoot fresh weight  (g) | |  | Shoot dry weight  (g) | |  | Plant height  (cm) | |
|  | 0 | 0.75% NaCl |  | 0 | 0.75% NaCl |  | 0 | 0.75% NaCl |
| WT | 2.06±0.11 | 1.02±0.06 |  | 0.32±0.02 | 0.18±0.01 |  | 27.31±2.55 | 18.62±0.46 |
| WPB106-cas-1 | 2.22±0.22 | 1.99±0.17** |  | 0.34±0.03 | 0.30±0.02** |  | 29.25±2.79 | 23.31±0.21** |
| WPB106-cas-2 | 2.20±0.23 | 2.15±0.20** |  | 0.34±0.02 | 0.29±0.04** |  | 29.12±2.38 | 23.94±0.30** |
| Significantly different from the performance of WT at ***P*<0.01. | | | | | | | | |
